# Identification of a Novel Elastin-Degrading Enzyme from the Fish Pathogen *Flavobacterium psychrophilum*

**DOI:** 10.1128/AEM.02535-18

**Published:** 2019-03-06

**Authors:** T. Rochat, D. Pérez-Pascual, H. Nilsen, M. Carpentier, S. Bridel, J.-F. Bernardet, E. Duchaud

**Affiliations:** a Virologie et Immunologie Moléculaires, INRA, Université Paris-Saclay, Jouy-en-Josas, France; b Norwegian Veterinary Institute, Bergen, Norway; c Institut Systématique Evolution Biodiversité, Sorbonne Université, Muséum National d’Histoire Naturelle, CNRS, EPHE, Paris, France; d Labofarm, Finalab, Loudéac, France; e Université de Versailles Saint-Quentin-en-Yvelines, Montigny-le-Bretonneux, France; Centers for Disease Control and Prevention

**Keywords:** *Flavobacterium psychrophilum*, elastase, elastin, fish pathogen, gluzincin, metalloprotease

## Abstract

Hydrolytic extracellular enzymes degrading host tissues potentially play a role in bacterial pathogenesis. Flavobacterium psychrophilum is an important bacterial pathogen of salmonid fish reared in freshwater throughout the world. Diversity among isolates has been described at the phenotypic, serological, and genomic levels, but the links between these various traits remain poorly understood. Using a genome-wide association study, we identified a gene encoding a novel elastinolytic enzyme in F. psychrophilum. To formally demonstrate enzymatic activity, this gene (*FP0506* from strain JIP 02/86) was expressed in the elastinolysis-deficient strain OSU THCO2-90, resulting in proficient elastin-degrading cells. The encoded protein is predicted to be a cell-surface-exposed lipoprotein with no homology to previously reported elastases. FP0506 might belong to the zincin tribe and gluzincin clan of metalloproteases, and this new elastase-encoding gene seems to be present only in some members of the family *Flavobacteriaceae*.

**IMPORTANCE** Elastin is an important proteinaceous component of vertebrate connective tissues (e.g., blood vessels, lung, and skin), to which it confers elasticity. Elastases have been identified in a number of pathogenic bacteria. They are thought to be required for tissue penetration and dissemination, acting as “spreading factors.” Flavobacterium psychrophilum is a devastating bacterial pathogen of salmonid fish (salmon and trout) that is responsible for severe economic losses worldwide. This pathogen displays strong proteolytic activities. Using a variety of techniques, including genome comparisons, we identified a gene encoding a novel elastase in F. psychrophilum. The encoded protein is predicted to be a cell-surface-exposed lipoprotein with no homology to previously reported elastases. In addition, this elastase likely belongs to a new family of proteases that seems to be present only in some members of this important group of bacteria.

## Abstract (precis)

Elastin is an important proteinaceous component of vertebrate connective tissues (e.g., blood vessels, lung, and skin), to which it confers elasticity. Elastases have been identified in a number of pathogenic bacteria. They are thought to be required for tissue penetration and dissemination, acting as “spreading factors.” Flavobacterium psychrophilum is a devastating bacterial pathogen of salmonid fish (salmon and trout) that is responsible for severe economic losses worldwide. This pathogen displays strong proteolytic activities. Using a variety of techniques, including genome comparisons, we identified a gene encoding a novel elastase in F. psychrophilum. The encoded protein is predicted to be a cell-surface-exposed lipoprotein with no homology to previously reported elastases. In addition, this elastase likely belongs to a new family of proteases that seems to be present only in some members of this important group of bacteria.

## INTRODUCTION

Extracellular enzymes that are able to degrade the structural barriers of the host are often considered significant virulence factors of pathogens. Secreted proteases that damage host tissues have been identified in some pathogenic bacteria and have been shown to play roles in the pathogenicity of the organisms. These enzymes are thought to be required for tissue penetration and dissemination, and the term “spreading factors” is sometimes used to refer to these enzymes collectively (1). Elastin is an extracellular matrix protein responsible for the elastic properties of vertebrate tissues. It is mainly composed of hydrophobic amino acids such as glycine and proline, and it forms fibers through extensive protein cross-linking in connective tissue. Elastinolytic enzymes have been identified in some pathogenic bacteria and fungi, including Leptospira interrogans, Pseudomonas aeruginosa, Vibrio vulnificus, Dichelobacter nodosus, and Aspergillus fumigatus (references 2–5 and references therein). Bacterial elastases facilitate the invasion of the host, provoking hemorrhage, tissue necrosis, and increased vascular permeability, as reported for the protein VvpE of V. vulnificus (4). Elastases can also contribute to immune evasion, as shown for LasB of P. aeruginosa (6).

Flavobacterium psychrophilum, a bacterium belonging to the family *Flavobacteriaceae*, is a serious bacterial pathogen of salmonid fish reared in freshwater throughout the world (7, 8). The two main clinical forms of the disease are rainbow trout fry syndrome and bacterial cold water disease (BCWD) (9). The clinical signs of BCWD are severe erosions of the caudal fin and peduncle and skin ulcers associated with destruction of the muscle, often localized at the base of the dorsal fin (saddleback disease). Outbreaks often result in considerable economic losses (10, 11). Phenotypic, serological, and genomic diversity among isolates has been described, but the links between these different areas remain largely unexplored (12–15). It also remains unclear how the respective contributions of the pathogen characteristics, as opposed to the susceptibility of the fish host, affect the success and severity of the infection.

Since the first attempts to investigate the physiological properties of this bacterium several decades ago, the high proteolytic capacity of the bacterium has been observed in *in vitro* assays, suggesting that at least certain strains produce extracellular enzymes that degrade components of fish skin, muscle, and cartilage, contributing to the clinical signs observed (16). Differences between strains in their ability to degrade specific protein substrates have been reported. Most isolates produce enzymes that are able to degrade casein or gelatin, whereas the ability to digest collagen and elastin is restricted to some isolates (17–19). Moreover, Madsen and Dalsgaard suggested that elastin-degrading isolates of F. psychrophilum appeared to be more virulent than isolates devoid of elastinolytic activity (20). Soule et al. reported that elastin-hydrolyzing ability was most closely associated with some genetic lineages (21). However, no elastase-encoding gene has been characterized to date, and genome mining failed to identify a *bona fide* elastase gene in F. psychrophilum genomes (12, 18, 22–25).

In the present study, we used a genome-wide association approach to identify the gene responsible for this enzymatic activity. We formally demonstrated the protein function through gene expression in an elastinolysis-deficient F. psychrophilum strain, and we produced *in silico* evidence that the protein likely belongs to the gluzincin clan of metalloproteases (MPs), which is restricted to some members of the family *Flavobacteriaceae*.

## RESULTS

### Comparative genomics identifies a gene encoding an elastinolytic enzyme in F. psychrophilum.

**FIG 1:**
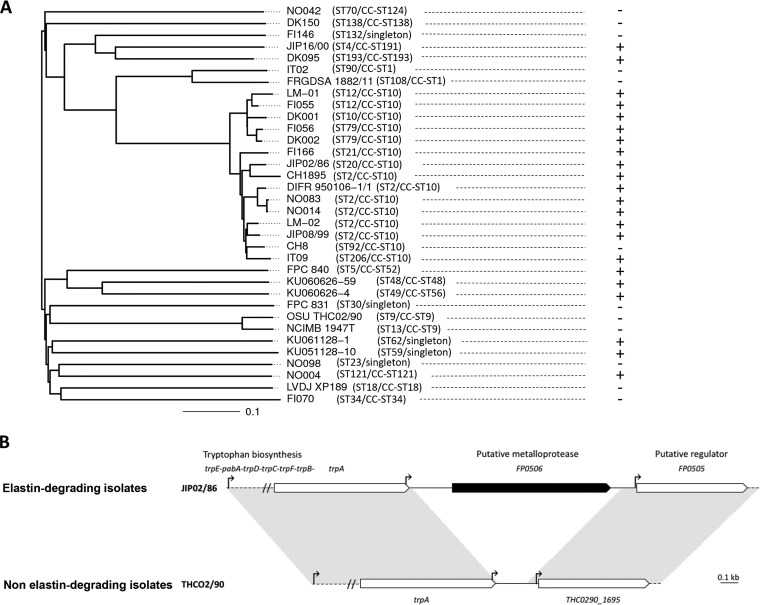
(A) Relationships between phylogeny and elastin-degrading capacity for the 34 F. psychrophilum strains used in this study. The presence (+) or absence (−) of elastin hydrolysis, observed as a clear halo surrounding the bacterial colonies on TYES agar enriched with 0.75% elastin, is indicated. The ST and CC of the strains are indicated in parenthesis. The phylogenetic relationships were captured in a cgMLST tree (12). (B) Comparison of genomic organization of the elastin-encoding gene neighborhood in elastin-degrading strains and non-elastin-degrading strains. White arrows represent core genome genes (i.e., genes conserved in the whole-genome data set for which deduced proteins display >80% identity with >80% coverage); the black arrow represents the predicted elastinolytic-encoding gene. Vertical arrows indicate putative motifs corresponding to the consensus sequence of *Bacteroidetes* promoters (TAnnTTTG). No significant rho-independent terminator was identified using the ARNold software for finding terminators (http://rna.igmors.u-psud.fr/toolbox/arnold).

The elastin degradation assay was performed with 34 F. psychrophilum strains (Table 1; also see Fig. S1 in the supplemental material) for which genome sequences were available (12). This analysis revealed that 22 of the 34 strains were proficient in elastin degradation (Fig. S1). Strikingly, elastin degradation ability did not follow exactly the phylogeny based on core genome multilocus sequence typing (cgMLST) (Fig. 1A). For example, strain CH8, which belongs to clonal complex (CC)-sequence type 10 (ST10), is deficient in elastin degradation, whereas all of the other strains belonging to this CC are proficient in this activity. Comparison of the genome contents of these strains revealed a unique gene that was present in all elastin-degrading isolates (*n* = 22) and absent in all non-elastin-degrading isolates (*n* = 12). When present, this gene (e.g., *FP0506* from strain JIP 02/86) is encompassed in a set of orthologous genes displaying a conserved organization and located between highly conserved core genome genes (i.e., the *trp* operon encoding enzymes of the tryptophan biosynthesis pathway and a gene encoding a TetR family transcriptional regulator of unknown function) (Fig. 1B). To determine the transcriptional structure of the locus, we searched expression signals *in silico* and performed reverse transcriptase PCR (RT-PCR) experiments. A motif corresponding to the *Bacteroidetes*-specific promoter sequence TAnnTTTG (26) was identified for *FP0506*, indicating that transcription may be initiated ∼250 bp upstream of its start codon. This putative promoter is enclosed in the coding sequence of *trpA*, the last gene of the *trp* operon (Fig. 1B). Because no Rho-independent terminator was identified in the *trpA-FP0506* intergenic region, *FP0506* could also be cotranscribed with the *trp* operon. RT-PCR experiments using overlapping primer sets showed that *trpA* and *FP0506* were part of the same transcript in strain JIP 02/90 (Fig. S2). Using cDNA from an elastase-negative strain (OSU THCO2-90), we showed that cotranscription occurred between *trpA* and the TetR family transcriptional regulator gene in the absence of *FP0506*, confirming the absence of an efficient terminator in this intergenic region.

**TABLE 1:** Bacterial strains, plasmids, and oligonucleotides used in this study

Plasmid, strain, or primer	Description or sequence (5′ to 3′)^*a*^	Source or reference
Plasmids		
pCP*Gm*^r^	E. coli-F. psychrophilum shuttle plasmid; ColE1 ori (pCP1 ori), Ap^r^ (Gm^r^)	29
pCP*Gm*^r^-*FP0506*	pCP*Gm*^r^ derivative carrying the FP0506 gene; Ap^r^ (Gm^r^)	This study
E. coli strains		
S17-1	*recA pro hsdR* RP4-2(Tc^r^::Mu-Km^r^::Tn*7* Str^r^)	58
MFD*pir*	MG1655 RP4-2-Tc::[ΔMu1::*aac(3)IV*-Δ*aphA*-Δ*nic35*-ΔMu2::*zeo*] Δ*dapA*::(*erm-pir*) Δ*recA*	45
F. psychrophilum strains		
FRGDSA 1882/11	Isolated from Oncorhynchus mykiss (France)	12
CH1895	Isolated from Salmo trutta (Switzerland)	12
CH8	Isolated from O. mykiss (Switzerland)	12
DK001	Isolated from O. mykiss (Denmark)	12
DK002	Isolated from O. mykiss (Denmark)	12
DK095	Isolated from Gasterosteus aculeatus (Denmark)	12
DK150	Isolated from O. mykiss (Denmark)	12
FI055	Isolated from O. mykiss (Finland)	12
FI056	Isolated from O. mykiss (Finland)	12
FI070	Isolated from Perca fluviatilis (Finland)	12
FI146	Isolated from pond water (Finland)	12
FI166	Isolated from Salmo salar (Scotland)	12
FPC 831	Isolated from Oncorhynchus kisutch (Japan)	12
FPC 840	Isolated from Plecoglossus altivelis (Japan)	12
IT02	Isolated from O. mykiss (Italy)	12
IT09	Isolated from O. mykiss (Italy)	12
JIP 02/86	Isolated from O. mykiss (France)	18
JIP 08/99	Isolated from O. mykiss (France)	12
JIP 16/00	Isolated from O. mykiss (France)	12
KU 051128-10	Isolated from river water (Japan)	12
KU 060626-4	Isolated from P. altivelis (Japan)	12
KU 060626-59	Isolated from P. altivelis (Japan)	12
KU 061128-1	Isolated from river water (Japan)	12
LM-01-Fp	Isolated from O. mykiss (Chile)	12
LM-02-Fp	Isolated from O. mykiss (Chile)	12
LVDJ XP189	Isolated from Tinca (France)	12
NCIMB 1947^T^	Isolated from O. kisutch (USA)	59
NO004	Isolated from S. trutta (Norway)	12
NO014	Isolated from O. mykiss (Norway)	12
NO042	Isolated from S. salar (Norway)	12
NO083	Isolated from O. mykiss (Norway)	12
NO098	Isolated from S. salar (Norway)	12
DIFR 950106-1/1	Isolated from O. mykiss (Denmark)	12
OSU THCO2-90	Isolated from O. kisutch (USA)	17
TRV103	OSU THCO2-90 *gldG*::Tn*4351* (Em^r^)	29
TRV272	OSU THCO2-90 pCP*Gm*^r^ (Gm^r^)	29
TRV300	OSU THCO2-90 pCP*Gm*^r^-*FP0506* (Gm^r^)	This study
TRV392	OSU THCO2-90 *gldG*::Tn*4351* pCP*Gm*^r^-*FP0506* (Em^r^, Gm^r^)	This study
Oligonucleotides		
TRO350	AATTATCCTTTTTATTATCCCTCAAA	
TRO351	TTATAGGGAATTCCGGACCG	
TRO352	TTTGAGGGATAATAAAAAGGATAATTGGAGCCATTATTGGAAGTGC	
TRO353	CGGTCCGGAATTCCCTATAACATTAAAATCGGTCATGGGTATT	
TRO137	GAGGGAACGACGCAAAGCGATAGTTC	
TRO138	GGAAACAGCTATGACCATGATTACGCC	
TRO383	ACCCCACAAACATCTAATGAGC	
TRO384	CTTAAAACAACCAAACAGTGTTTTTTACCG	
TRO385	ATAATAATTGGGCATCAGCTGCAA	
TRO387	AGATAATCTGCCAATCTGCGG	
TRO388	GCGTCAAAATTAGCTGGTTTAGC	
TRO389	GTCGGCTGTTGTTAAAATGGGT	
TRO442	TTGCAGCTGATGCCCAATTATTAT	

a Underlined sequences in Gibson assembly primers correspond to regions that hybridize with the elastase-encoding gene. Ap^r^, ampicillin resistance; Tc^r^, tetracycline resistance; Km^r^, kanamycin resistance; Str^r^, streptomycin resistance; Em^r^, erythromycin; Gm^r^, gentamicin resistance.

### Heterologous expression of FP0506 in an elastase-deficient F. psychrophilum strain results in elastin degradation.

**FIG 2:**
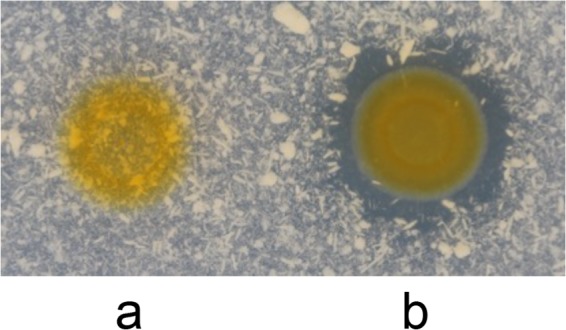
Heterologous expression of *FP0506* in strain OSU THCO2-90, an elastinolysis-deficient F. psychrophilum isolate. Proteolytic activity was assayed by plating bacterial cultures on TYES agar supplemented with 0.75% (wt/vol) elastin. Observation of clear halos was recorded after 5 days of incubation at 18°C. Strain OSU THCO2-90 carried the empty plasmid pCP*Gm*^r^ (a) or pCP*Gm*^r^-*FP0506* (b).

To demonstrate formally that *FP0506* encodes an elastase, the gene was cloned into the replicative plasmid pCP*Gm*^r^ and introduced by conjugation into the elastinolysis-deficient strain OSU THCO2-90, and the proteolytic activity assay was performed by spotting bacterial cultures on tryptone-yeast extract-salts (TYES) plates supplemented with elastin. In contrast to the strain carrying the empty plasmid, the presence of pCP*Gm*^r^-*FP0506* resulted in a clearing area around the bacterial growth, indicating strong degradation of elastin (Fig. 2). This experiment clearly demonstrated that gene *FP0506* encodes an elastase. Due to the apparent diffusion of proteolytic activity on solid medium observed in *in vitro* assays, we hypothesized that FP0506 could be released into the external milieu. Moreover, differences in diffusion ability among elastin-degrading strains, resulting in different halo sizes, were noticed (Fig. S1). Because promoter regions of the *FP0506* locus are conserved between elastase-positive isolates, these variations are likely unrelated to a transcriptional modulation effect but may arise from differences in elastase release between isolates.

### FP0506 encodes a putative cell-surface-exposed lipoprotein that likely belongs to a new MP family of the gluzincin clan.

The deduced FP0506 protein is 296 amino acids long and was originally annotated as a probable MP precursor, but that annotation was based on poor BLASTp scores (18). Strikingly, FP0506 does not display any significant primary homologies with known elastases. Nevertheless, FP0506 possesses a strong lipoprotein signal prediction score, with a cleavage site located between residues 21 and 22. In addition, the amino acid residues located downstream of the invariant first cysteine (+1 C of the mature protein) likely encode the lipoprotein export signal that was recently reported for the phylogenetically related bacterium Capnocytophaga canimorsus (27). Indeed, the +3 lysine, +5 aspartic acid, and +6 aspartic acid residues, reported to be present in 72%, 44%, and 11%, respectively, of the surface-exposed lipoproteins of C. canimorsus, are conserved in FP0506 (Fig. S4). No carboxyl-terminal domain belonging to the TIGR04183 or TIGR04131 family (28) was predicted, suggesting that FP0506 is not secreted by the type IX secretion system (T9SS). Therefore, it may be predicted that the FP0506 mature protein is 275 amino acids long and is located at the cell surface, facing the external milieu. However, this putative localization does not fit with the diffusible proteolytic activity observed *in vitro* for most of the isolates. In order to determine whether the lipid-anchored form of FP0506 is subjected to a second processing event through the action of an external protease, *FP0506* was expressed in a F. psychrophilum OSU THCO2-90 mutant found to be deficient in exoproteolytic activities (29). Indeed, the inactivation of *gldG* is suspected to affect the T9SS machinery indirectly, resulting in protein secretion deficiency. The introduction of pCP*Gm*^r^-*FP0506* into the *gldG*::Tn4351 mutant resulted in a diffusible elastin-degrading ability, as observed for the wild-type strain (Fig. S5). This result suggests that neither the T9SS nor the T9SS-dependent secreted proteases are required for the release of elastase.

In an attempt to classify FP0506 rationally, we used a number of bioinformatic tools, aiming to identify additional protein motifs, conserved residues, and folds. A motif search identified the triad HExxH (amino acid positions 144 to 148 in the full-length protein), which may account for MP prediction. This short HExxH motif includes two metal-binding histidines and a glutamate that harbors the catalytic function. Moreover, MPs contain additional residues that extend beyond the HExxH motif (also known as the Jongeneel consensus sequence), i.e., the absence of any charged amino acids (other than the two histidines and the glutamate) within a 10-amino-acid stretch and the presence of a hydrophobic residue 2 positions after the second histidine (30, 31). FP0506 perfectly matches these criteria (Fig. S4). Accordingly, FP0506 should belong to clan MA of the MPs. This clan contains a variety of MPs, which can be divided into more than 100 families according to the MEROPS classification (version 11) (32). Very weak, partial, and poorly significant similarities to different families belonging to the MA clan (e.g., families M7, M12, and M78) were detected. Therefore, it was impossible to assign FP0506 properly to any MA clan peptidase family. According to the classification proposed by Cerdà-Costa and Gomis-Rüth (33), proteins encompassing the HExxH motif are grouped into the zincin tribe, which is subdivided into different clans according to the presence of additional residues. The two main zincin clans are the metzincins and gluzincins. FP0506 does not possess the metzincin-specific residues and therefore does not belong to this clan. In addition to and downstream of the HExxH motif, however, we noticed an ExxA motif (positions 167 to 170), containing invariant residues, across the entire homologous protein set (see below). This highly conserved motif suggests that FP0506 may belong to the gluzincin clan.

Because of the remote primary homologies mentioned previously, we applied HHpred, which uses hidden Markov model (HMM)-HMM comparisons (after signal peptide removal, to increase the signal/noise ratio), to predict additional structures that might be relevant to assigning FP0506 to a peptidase clade/family. A HHpred search with the Pfam database (V31.0), encompassing a large collection of protein families, identified (i) PF04228.12, corresponding to a putative neutral zinc metallopeptidase, as the first hit (E value of 1.8e−19) and (ii) PF01435.17, corresponding to peptidase family M48, as the second hit (E value of 1.7e−14), also suggesting that FP0506 belongs to the gluzincin clan. This clan is divided into several families based on additional characteristics. However, it was not possible to determine a unique gluzincin family for FP0506 by relying only on the presence of additional specific motifs.

### Structural prediction.

In order to build a structural model for FP0506, we used HHpred to search the three-dimensional (3D) protein structures in the Protein Data Bank (PDB) (PDB_mmCIF70_28_Jul) and identified 3C37 as the best protein template candidate (Fig. S6). This PDB entry corresponds to a putative Zn-dependent peptidase (UniProtKB accession no. Q74D82) from Geobacter sulfurreducens, which is in good agreement with our previous primary homology predictions. In addition, Q74D82 is a lipoprotein of about the same size (253 amino acids) as FP0506. Even though the pairwise alignment obtained with HHpred revealed a low primary identity percentage (17% over 65% of the length of the mature FP0506 protein), the result was highly significant (probability, 99.54%; E value, 4e−16). Of utmost importance, the previously predicted gluzincin motifs of FP0506 are correctly arranged in the alignment, i.e., the HExxH motif (positions 125 to 129 in Q74D82) and the ExxA motif containing the third zinc ligand, corresponding to the glutamate residue (position 181 in Q74D82) and the alanine residue (position 184 in Q74D82). The next three hits of HHpred were transmembrane proteins of the M48A family, likely of much lower significance. The same results were obtained with other structure prediction tools, i.e., Robetta, RaptorX, and I-Tasser. The best hit was always PDB 3C37, with 3D prediction values slightly above the threshold, while the next hits corresponded to peptidases belonging to different families, with very poor prediction scores.

**FIG 3:**
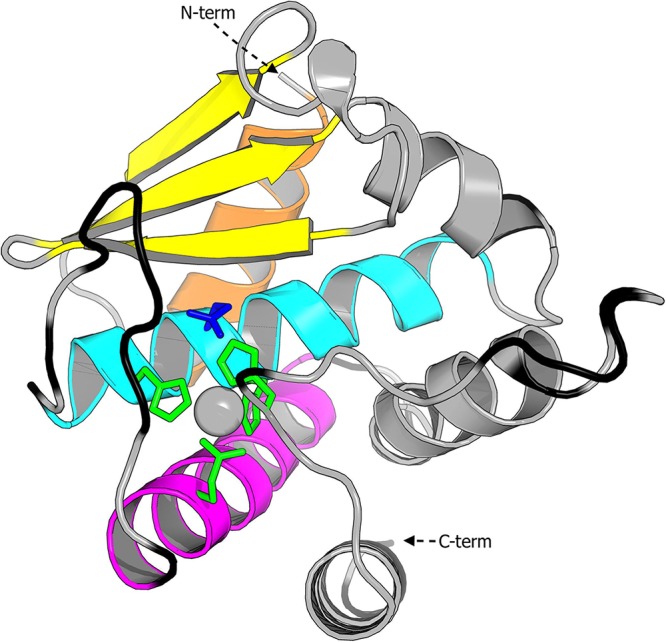
Three-dimensional model generated by Swiss-Model for FP0506 with PDB 3C37 as a template in a standard orientation (42). Regions with local QMEAN scores below 0.6 (local reliability threshold) are shown in black. The active site cleft is in the center. Features characteristic of gluzincin, including the active helix (cyan), the glutamate helix (magenta), the backing helix (orange), and the three beta sheets (yellow), are highlighted. The predicted four zinc-binding amino acids are in green, and the active glutamate is in dark blue. The metal atom is represented as a gray sphere.

Therefore, we used the 3C37 structure as a template to model the 3D structure of FP0506. The model was built with Swiss-Model, which predicted the structure of the aligned regions, i.e., residues 74 to 229 of FP0506 (Fig. 3). The overall score for the model (QMEAN, −3.64) was slightly above the reliability threshold of −4.0 (34–36). However, this rather low prediction value was mainly due to low local scores below the threshold (Fig. S7A and black regions in Fig. 3), which mostly corresponded to predicted protein loops that were not resolved in the original structure of 3C37. Of importance, the local scores were significant and above the threshold for the important secondary structures and critical residues identified in Q74D82, i.e., the zinc-binding amino acids (green residues in Fig. 3), the active glutamate (dark blue residue in Fig. 3), and the metal atom (gray sphere in Fig. 3), as well as the three beta sheets (yellow arrows in Fig. 3) and the backing helix (orange helix in Fig. 3). Therefore, the predicted global fold of FP0506 was in rather good agreement with the X-ray structure of Q74D82, and the critical residues perfectly fit the 3D atomic repartition (Fig. S7B). We also identified the structural features of the gluzincin clan, including the active site helix (cyan helix in Fig. 3) and the glutamate helix (magenta helix in Fig. 3).

### FP0506-homologous proteins are restricted to members of the family *Flavobacteriaceae*.

**FIG 4:**
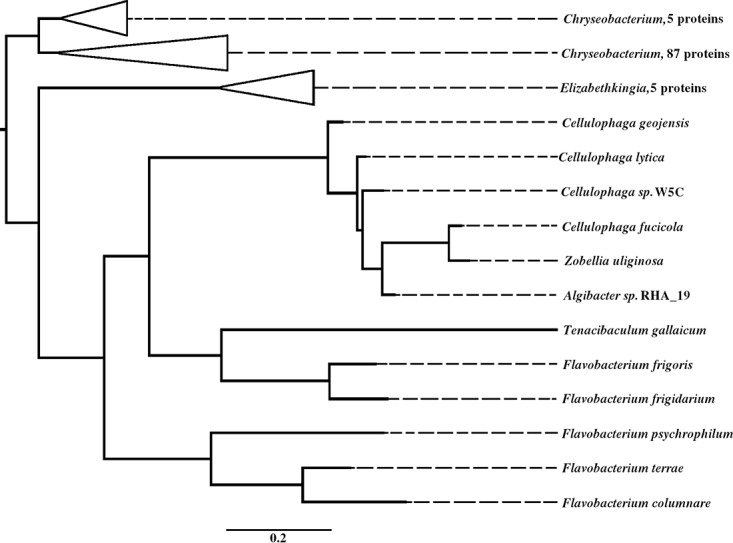
Phylogenetic tree of FP0506-homologous proteins. For the genera *Chryseobacterium* and *Elizabethkingia*, the branches have been collapsed for better reading. A complete phylogenetic tree, including accession numbers, is available in Fig. S8 in the supplemental material.

In order to identify proteins similar to FP0506, we used BLASTn with the nonredundant protein database. We used rather relaxed parameters (40% sequence identity and 80% query sequence coverage) and retrieved 109 proteins; all were predicted lipoproteins, and all were from bacteria belonging to the family *Flavobacteriaceae*. Among those, a number of pathogens were identified, such as Flavobacterium columnare (another important fish pathogen), Elizabethkingia meningoseptica and Elizabethkingia anophelis (two deadly human pathogens), Chryseobacterium indologenes (a less severe human-pathogenic bacterium), and a number of alga-degrading bacteria such as Zobellia galactanivorans, Zobellia uliginosa, and *Cellulophaga* species. The phylogenetic tree obtained with these sequences globally fits the tree obtained with 16S rRNA gene sequences, suggesting little horizontal gene transfer between these species (Fig. 4; also see Fig. S8).

In a search against the *Tara* Oceans data collection, a unique significant hit was obtained with contig TARA_076_MES_0.45-0.8_C13306631_1. This sequence was acquired from a water sample obtained at a depth of 800 m from a mesopelagic zone in the South Atlantic Ocean (*Tara* station 076). In a search of metagenome data sets using the MGnify tool at EMBL-EBI, the best hit (MGnify protein identification MGYP000403850383) also corresponded to a deep sea metagenome sample (EMBL accession no. SRR3963982). The corresponding protein sequence was highly similar (97% identity) to the one from the *Tara* Oceans collection. The second best hit (MGnify protein identification MGYP000672683253) corresponded to a human stool sample (EMBL accession no. ERS473408), the third hit (MGnify protein identification MGYP000518824090) to a sample from an atopic dermatitis human skin microbiome (EMBL accession no. SRR1950762), and the fourth hit (MGnify protein identification MGYP000270009725) to a human oral metagenome sample (EMBL accession no. SRR3735413). All of these sequences belonged to predicted lipoproteins of about the same size as FP0506 and all previously mentioned important residues and motifs were conserved, suggesting that these proteins are *bona fide* homologues of FP0506.

## DISCUSSION

In the pioneering works on F. psychrophilum in the 1980s, elastinolytic strains were reported (16). Elastin-degrading ability was associated with some genetic lineages (21), and elastin-degrading isolates of F. psychrophilum seemed to be more virulent than those devoid of elastase activity (20). Despite many attempts, however, no elastin-degrading enzyme was identified or cloned from F. psychrophilum (12, 18, 22–25). In this study, we took advantage of the availability of the genome sequences of 34 F. psychrophilum strains (12). By testing these strains for elastin degradation, we identified a single gene (*FP0506*) that was present exclusively in the elastin-degrading isolates. Gene *FP0506* from strain JIP 02/86 was cloned and expressed in the elastinolysis-deficient strain OSU THCO2-90, resulting in proficient elastin degradation of recipient cells. Biochemical studies would be necessary to determine the substrate specificity of elastin-degrading FP0506 protein toward other host proteins.

Among F. psychrophilum isolates proficient in elastin degradation, differences in halo size were noticed (Fig. S1). Some strains (e.g., FPC 840, NO004, and DK002) displayed almost no diffusion and elastin hydrolysis was restricted to the area beneath the bacterial growth, while other strains (e.g., NO083 and JIP 02/86) displayed a large halo. All strains (except CH08) belonging to CC-ST10 were able to degrade elastin, and most of them displayed a large halo. This finding suggests that elastin-degrading ability may provide a selective advantage for this epidemic, rainbow trout-associated group of isolates (12, 37). It was also the case for the ayu-associated strains (i.e., FPC 840, KU 060626-4, and KU 060626-59), as all 3 of the strains were proficient in elastin degradation. Conversely, the 2 rainbow trout-associated strains belonging to ST90 (i.e., IT2 and FRGDSA 1882/11) and the 3 Coho salmon-associated strains (i.e., OSU THCO2-90, FPC 831, and NCIMB 1947^T^) were devoid of elastinolytic activity. Therefore, it seems that there is a general tendency linking elastinolytic ability and strain genotype, as suggested previously (21). It would be of interest to extend this analysis to additional isolates in order to perform sound statistical correlations among strain genotype, host fish, and virulence.

In addition, because of differences in the extent of proteolysis diffusion on elastin-enriched agar, it may be suggested that elastin-degrading proteins might be released (at least to some extent) into the external milieu. Because of the surface-anchored, lipoproteic nature of this protein, this counterintuitive behavior suggests an active releasing process. Cloning of *FP0506* into a F. psychrophilum T9SS-defective mutant (29) still resulted in diffusible elastin-degrading ability, similar to findings for the wild-type genetic background (Fig. S5). This result suggests that an active T9SS is not required for the observed diffusion ability. Therefore, strain-dependent bacterial cell lysis (at least partial), outer membrane vesicle production, or another T9SS-independent releasing mechanism might explain the different halo sizes observed among strains.

Because the elastase-encoding gene is located at the same position in the genomes of all elastinolysis-proficient F. psychrophilum strains and because the neighboring regions are highly conserved, the distribution within the species likely results from intraspecies recombination. This recombinational behavior was observed previously on the genome scale (37) and was quantified recently (12). In addition, recombination has been suspected to be the driving force of serotype diversity in F. psychrophilum through gene shuffling at another locus encompassing genes involved in exopolysaccharide synthesis (38). It may be concluded that recombination-mediated gene gain and loss lead to some phylogenetic incongruities (e.g., strain CH8 lacks the elastin-encoding gene, whereas all other strains in CC-ST10 possess it).

Using RT-PCR experiments, we showed that the elastase was coexpressed with the *trp* operon and that cotranscription occurred between *trpA* and the TetR family transcriptional regulator gene in the absence of *FP0506*. As a result, *FP0506* is likely transcribed from its own promoter as monocistronic mRNA and as polycistronic mRNA together with the genes encoding proteins for tryptophan synthesis. The biosynthetic pathway for tryptophan is posttranscriptionally controlled by a leader transcript in several bacteria. In Escherichia coli, it is composed of a Trp-enriched short peptide and secondary structures that act as termination or antitermination elements, allowing correlation of transcription of the operon with the charged tRNA^Trp^ concentration. Interestingly, a similarly structured RNA sequence named *Bacteroid*-Trp RNA was identified *in silico* in *Bacteroidetes* species (39). This element is conserved upstream of the *trp* operon in F. psychrophilum, indicating that cells facing amino acid starvation may conjointly express the biosynthetic pathway for Trp, a rare amino acid in nature, and the elastinolytic protease that could liberate amino acids and peptides to be used as bacterial nutrients.

FP0506 does not display any significant homologies with known elastases. Because of remote similarities with several MPs belonging to different families, discrepancies regarding the prediction results were noticed, as follows: (i) BLASTp results were poorly informative, even with the MEROPS database dedicated to peptidases; (ii) InterProScan predicted only the lipoprotein secretion signal (positions 1 to 22), in perfect agreement with the results obtained with LipoP; and (iii) the COGnitor unique prediction was COG2321, a member of the Zn peptidase superfamily cl19825. In addition, the latter homology encompassed only 101 amino acid residues, corresponding to one-third of the full-length protein. Therefore, we used HHPred, which is based on pairwise comparisons of HMM profiles and is presumed to be more sensitive than the previously mentioned tools (40, 41). Indeed, HHPred was run using the FP0506 protein as a query (after signal peptide removal) with the Pfam-A (version 31.0) and PDB_mmCIF70 (version 28) databases. Significant results were obtained using both databases, suggesting that the FP0506 protein belongs to the zincin tribe and gluzincin clan of MPs (33).

Regardless of the prediction tool used (Swiss-Model, Robetta, RaptorX, or I-Tasser), the only slightly similar 3D structure was PDB 3C37, which corresponds to a putative Zn-dependent peptidase (UniProtKB accession no. Q74D82). According to UniProt, Q74D82 is annotated as being in the peptidase M48 family but with the lowest annotation score, which corresponds to an unreviewed status. In addition, the M48 peptidase family mostly contains transmembrane proteins, which is not the case for FP0506. Moreover, PDB 3C37 corresponds to an unpublished Structural Genomics Consortium target, and its exact function and proper classification have not been reported. However, Q74D82 is also a lipoprotein of about the same size (253 amino acids) as FP0506. Using predictive folding tools, we identified in FP0506 the 3D structural features of the gluzincin clan of MPs (33). The active site cleft divides the protein into two subdomains. The N-terminal subdomain (upper part of Fig. 3, as viewed in the standard MP orientation [42]) is above the cleft and contains three beta sheets, the backing helix, and the active site helix, which provides two metal-binding residues and the catalytic glutamate. The C-terminal subdomain is below the cleft and contains the glutamate helix, which runs parallel to the active site helix in a horizontal projection and provides the third metal-binding residue (i.e., glutamate at position 167). This residue is 3 positions upstream of the residue of the Ser/Gly turn (i.e., alanine at position 167), which is the most widespread residue among the gluzincin families. PDB 3C37 may contain a fourth metal-binding residue (histidine at position 227 of Q74D82), coming from the distal part of the C-terminal subdomain that is conserved in FP0506. This additional histidine is not a canonical feature of the gluzincin clan and appears to block the cleft, suggesting that PDB 3C37 is a nonfunctional protein. In the absence of additional data on PDB 3C37, crystallization artifacts cannot be ruled out, as suggested by Lopéz-Pelegrín et al. (43). Alternatively, these proteins may correspond to a zymogen with a fourth zinc-blocking residue, as reported for PDB 3KHI of Klebsiella pneumoniae (44). Within the gluzincin clan, three families harbor the HExxH and ExxA motifs. Accordingly, FP0506 might belong to the M48/M56 intramembrane MP family, the anthrax lethal factor family, or the neprilysin family or to an undescribed protein family.

All FP0506-homologous proteins identified using the NCBI nonredundant database were members of the family *Flavobacteriaceae*. Most were identified in marine members of the family, while others were retrieved from pathogenic representatives. Intriguingly, some FP0506-homologous proteins were identified in alga-degrading bacteria, such as some *Zobellia* specie*s*. It is possible that these bacteria experience living stages during which they are associated with elastin-possessing organisms (e.g., marine invertebrates) instead of algae, and FP0506-homologous proteins (such as ZGAL_1019) might be required for elastin degradation in this context. A single, unique, FP0506 homologue was identified in the *Tara* Oceans data collection. This unique hit was rather surprising, because this collection encompasses 243 ocean microbiome whole-genome sequencing assemblies and 111,530,851 predicted genes. One possible explanation could be that *FP0506*-homologous genes belonging to bacteria that do not occur in the water column but are attached to some marine animals have passed unnoticed, since such ecological niches were not sampled during the *Tara* expedition. Intriguingly, in searches using other metagenome data sets, only very limited numbers of FP0506-homologous proteins were identified. The best hit using the MGnify tool also corresponded to a deep sea metagenome sample. The sequence was 97% identical to that from the *Tara* Oceans data collection, suggesting that the two belong to the same or highly related taxonomic groups of bacteria. Finally, the next three significant hits corresponded to human microbiome samples (i.e., stool, skin, and oral cavity samples), suggesting that human-associated bacteria, as observed previously for some *Elizabethkingia* and *Chryseobacterium* species, do possess this gene. Therefore, we propose that FP0506 and homologous proteins are likely required by some pathogenic bacteria for efficient tissue penetration and dissemination, acting as spreading factors.

## MATERIALS AND METHODS

### Bacterial strains and growth conditions.

The strains, plasmids, and primers used in this study are listed in Table 1. Detailed data on the F. psychrophilum isolates (i.e., fish host, country and year of isolation, and culture collection) are available in Table S1 in the supplemental material. Escherichia coli strains S17-1 and MFD*pir* (45) were used for cloning and transfer of plasmid DNA into F. psychrophilum OSU THCO2-90 by conjugation. E. coli was grown at 37°C in Luria-Bertani (LB) medium supplemented with 0.3 mM diaminopimelic acid (Sigma-Aldrich Co.) if required, with 15 g of agar per liter added for solid medium. F. psychrophilum strains were grown at 200 rpm and 18°C in TYES broth (0.4% [wt/vol] tryptone, 0.04% yeast extract, 0.05% [wt/vol] MgSO_4_·7H_2_O, 0.02% [wt/vol] CaCl_2_·2H_2_O, 0.05% [wt/vol] d-glucose [pH 7.2]). Stock cultures were preserved at −80°C in TYES broth containing 20% (vol/vol) glycerol.

### Elastin degradation assay.

Proteolytic activity on solid medium was visualized by plating 10 µl of stationary-phase (48-h) bacterial culture on TYES agar supplemented with 0.75% (wt/vol) elastin from bovine neck ligament (product no. E1625; Sigma-Aldrich). The ability of F. psychrophilum strains to hydrolyze elastin was recorded after 4 to 10 days of incubation at 18°C, by observation of a zone of clearing surrounding the bacterial growth. Experiments were performed at least in duplicate.

### Genome comparisons.

Genome comparisons were performed using the web interface MicroScope (46), which allows graphic visualization enhanced by synchronized representation of synteny groups (http://www.genoscope.cns.fr/agc/mage). Comparison of the gene contents between bacterial strains was performed by pairwise proteome similarity searches using BLASTp bidirectional best hit and MicroScope default parameters (i.e., >80% protein identity and >80% coverage). Locus tags for elastase-encoding genes identified in F. psychrophilum were CH1895_90007 for strain CH1895, IB65_02450 for strain DIFR 950106-1/1, DK001_90007 for strain DK001, DK002_20007 for strain DK002, DK095_600033 for strain DK095, FI055_440007 for strain FI055, FI056_20048 for strain FI056, FI166_140049 for strain FI166, FPC840_310011 for strain FPC 840, IT9_150007 for strain IT09, FP0506 for strain JIP 02/86, JIP0899_430034 for strain JIP 08/99, JIP1600_330003 for strain JIP 16/00, KU05112810_20011 for strain KU051128-10, KU06062604_690011 for strain KU060626-4, KU06062659_80002 for strain KU060626-59, KU06112801_670011 for strain KU061128-1, LM01FP_50050 for strain LM-01, LM02FP_v1_80047 for strain LM-02, NO004_420038 for strain NO004, NO014_180047 for strain NO014, and NO083_50048 for strain NO083.

### Protein homology search and phylogenetic tree reconstruction.

Searches for elastase-homologous proteins were performed using FP0506 from strain JIP02/86 to query the nonredundant protein sequence database, the *Tara* Oceans data collection (http://bioinfo.szn.it/tara-blast-server), and the MGnify data collection (https://www.ebi.ac.uk/metagenomics) for microbiome data sets. Proteins with at least 40% sequence identity and 80% query sequence coverage were retrieved. Sequence alignment was performed using the MUSCLE tool (version 3.8.31) implemented in UGENE software (version 1.31) (47), and the resulting alignment was visualized. Sequence alignment was manually checked, and sequences displaying inconsistencies (i.e., errors in start site prediction) were discarded. Phylogenetic tree reconstruction was performed after peptide signal removal (to increase the signal/noise ratio), using neighbor joining and the Jones-Taylor-Thornton distance matrix model. UGENE software (47) was used for sequence alignment, curation, and tree building. Tree accuracy was measured by bootstrap resampling of 100 replicates. The resulting tree was drawn using FigTree (version 1.4.3) (http://tree.bio.ed.ac.uk/software/figtree). A cgMLST tree (Fig. 1) was built using 1,549 conserved single-copy genes. Unique identifiers were attributed to the distinct allele types defined on the basis of the multiple DNA sequence alignments, and a tree was built with the neighbor-joining approach, as reported by Duchaud et al. (12).

### Bioinformatic tools for protein sequence analysis, classification, and prediction of structural characteristics.

Lipoprotein signal prediction was performed using the LipoP 1.0 server (48). InterProScan 5 (49) was used to search the InterPro database (50). Clusters of Orthologous Groups (COG) assignment was performed using COGnitor (51). HHPred was run online (https://toolkit.tuebingen.mpg.de/#/tools/hhpred), using the MPI Bioinformatics Toolkit (40, 41). The structural models were realized with Swiss-Model (https://swissmodel.expasy.org) (52, 53), Robetta (http://robetta.bakerlab.org) (54), RaptorX (http://raptorx.uchicago.edu) (55), and I-Tasser (https://zhanglab.ccmb.med.umich.edu/I-TASSER) (56), with default parameters. The deduced structures were visualized with PyMOL.

### Total RNA extraction and RT-PCR.

Bacterial cells from 10-ml cultures of F. psychrophilum strains JIP 02/86 and OSU THCO2-90 (carrying the *FP0596* gene and not, respectively) were collected, at an optical density at 600 nm (OD_600_) of 1, by centrifugation for 3 min after the addition of 0.5 volume of frozen killing buffer (20 mM Tris-HCl [pH 7.5], 5 mM MgCl_2_, 20 mM NaN_3_) to the culture sample. Cell pellets were frozen in liquid nitrogen and stored at −80°C. For extraction, the pellets were resuspended in 800 μl of lysis buffer (4 M guanidine thiocyanate, 25 mM sodium acetate [pH 5.2], 5 g/liter *N*-laurylsarcosinate), immediately mixed with 800 µl hot acid-phenol (product no. P4682; Sigma-Aldrich), and incubated for 10 min at 65°C for efficient cell lysis. The aqueous phases were recovered after the addition of 400 µl chloroform and centrifugation at 16,000 × *g* for 10 min at room temperature. The samples were extracted at least three times with an equal volume of acid-phenol/chloroform/isoamyl alcohol (25:24:1 [pH 4.5]) and once with chloroform. Total RNAs were precipitated with ethanol, and the pellets were resuspended in RNase-free water. RNA extracts (50 µg) were treated with DNase I (Qiagen) to remove residual genomic DNA and then were purified using the RNA clean-up and concentration kit (Norgen Biotek). cDNA was synthesized using the SuperScript II reverse transcriptase kit (Invitrogen) and random primers. Negative-control reactions without reverse transcriptase were performed in parallel. Overlapping PCRs were performed between *FP0506* and flanking genes in strains JIP 02/86 and OSU THCO2-90, as described in Fig. S2A in the supplemental material. The primers used are listed in Table 1.

### Cloning of the FP0506 gene in the E. coli-F. psychrophilum shuttle vector pCP*Gm*^r^.

A 1.4-kb region encompassing the coding sequence of *FP0506* as well as 330 bp upstream and 170 bp downstream was introduced by Gibson’s assembly in the pCP*Gm*^r^ plasmid, which confers gentamicin resistance in F. psychrophilum (29). The plasmid map is available in Fig. S3. Briefly, FP0506 DNA was amplified from strain JIP 02/86 by PCR using primers TRO352 and TRO353. The vector was amplified by PCR using primers TRO350 and TRO351 and pCP*Gm*^r^ as a DNA matrix, as reported previously (29). The DNA assembly resulted in the insertion of *FP0506* upstream of the expression signals of open reading frame 1 of pCP1, a cryptic plasmid isolated from a F. psychrophilum strain (57). The resulting plasmid, pCP*Gm*^r^-*FP0506*, was transferred to E. coli MFD*pir* by electroporation and was verified by DNA sequencing. pCP*Gm*^r^-*FP0506* was then introduced into strain OSU THCO2-90 or a *gldG*::Tn4351 derivative mutant by conjugation, as described previously (29). Transconjugants were selected by plating on TYES agar supplemented with 10 or 50 µg/ml gentamicin, respectively, and incubation at 18°C for up to 5 days.

## Supplementary Material

Supplemental file 1
