# Supplementary material for: Identification of a Novel Elastin-Degrading Enzyme from the Fish Pathogen Flavobacterium psychrophilum
Source: Appl Environ Microbiol. 2019 Mar 6;85(6):e02535-18. doi: 10.1128/AEM.02535-18 (PMC6414381; doi:10.1128/AEM.02535-18)

Supplementary material

Identification of a novel elastin-degrading enzyme from *Flavobacterium psychrophilum*

T. Rochat[1], D. Pérez-Pascual[1,+], H. Nilsen[2], M. Carpentier[3], S. Bridel[1,4,5], J-F Bernardet[1], and E. Duchaud[1*]

Affiliations
[1] VIM, INRA, Université Paris-Saclay, 78350, Jouy-en-Josas, France.
[2] Norwegian Veterinary Institute, P.B. 1263 Sentrum, 5811 Bergen, Norway
[3] Institut Systématique Evolution Biodiversité (ISYEB), Sorbonne Université, MNHN, CNRS, EPHE, 45 rue Buffon, CP 50, 75005 Paris, France.
[4] Labofarm, Finalab, Loudéac, France
[5] Université de Versailles Saint-Quentin-En-Yvelines, Montigny-Le-Bretonneux, France
*corresponding author: E-mail: eric.duchaud@inra.fr
[+]present address: Département de Microbiologie, Unité de Génétique des Biofilms, Institut Pasteur, 25-28 rue du Dr. Roux, F-75015 Paris, France.

List of contents

Table S1. *F. psychrophilum* used for comparative genomics analysis.

Fig. S1. Elastinolytic activity of all 34 isolates on TYES agar supplemented with 0.75% elastin.

Fig. S2. RT-PCR analysis of the elastase locus in strains JIP 02/86 and OSU THCO2-90.

Fig. S3. Plasmid map of pCP*Gm*^{r}-*FP0506*.

Fig. S4. Predicted protein domains and amino-acid residues in FP0506.

Fig. S5. Expression of *FP0506* in a *F. psychrophilum* mutant deficient for exoproteolytic activity.

Fig. S6. Pairwise alignment of the mature FP0506 and PDB-3C37 obtained using HHpred.

Fig. S7. Predicted local quality score of FP0506 using the SwissModel and PDB-3C37 as a template.

Fig. S8. Phylogenetic tree of FP0506 homologous proteins.

Table S1. *F. psychrophilum* used for comparative genomics analysis

Strain	Genome accession number (reference)	Country	Host	year	Sequencing status	Strain provided by
FRGDSA 1882/11	GCA_900186415 (5)	France	*Oncorhynchus mykiss*	2011	draft	P. Daniel, Laboratoire Vétérinaire Départemental des Pyrénées et des Landes, Mont-de-Marsan, France
CH1895	GCA_900186435 (5)	Switzerland	*Salmo trutta*	2011	draft	N. Streppavara, Cantonal Institute of Microbiology, Bellinzona, Switzerland
CH8	GCA_900186345 (5)	Switzerland	*Oncorhynchus mykiss*	2009	draft	N. Streppavara, Cantonal Institute of Microbiology, Bellinzona, Switzerland
DK001	GCA_900186405 (5)	Denmark	*Oncorhynchus mykiss*	2009	draft	Culture collection of the National Veterinary Institute, Frederiksberg, Denmark
DK002	GCA_900186425 (5)	Denmark	*Oncorhynchus mykiss*	1990	draft	Culture collection of the National Veterinary Institute, Frederiksberg, Denmark
DK095	GCA_900186385 (5)	Denmark	*Gasterosteus aculeatus*	2000	draft	Culture collection of the National Veterinary Institute, Frederiksberg, Denmark
DK150	GCA_900186365 (5)	Denmark	*Oncorhynchus mykiss*	1995	draft	Culture collection of the National Veterinary Institute, Frederiksberg, Denmark
FI055	GCA_900186395 (5)	Finland	*Oncorhynchus mykiss*	1996	draft	Culture collection of Åbo Akademi University, Turku, Finland
FI056	GCA_900186375 (5)	Finland	*Oncorhynchus mykiss*	1996	draft	Culture collection of Åbo Akademi University, Turku, Finland
FI070	GCA_900186445 (5)	Finland	*Perca fluviatilis*	2006	draft	Culture collection of Åbo Akademi University, Turku, Finland
FI146	GCA_900186455 (5)	Finland	Pond water	2000	draft	Culture collection of Åbo Akademi University, Turku, Finland
FI166	GCA_900186565 (5)	Scotland	*Salmo salar*	2007	draft	Culture collection of Åbo Akademi University, Turku, Finland
FPC 831	GCA_900186485 (5)	Japan	*Oncorhynchus kisutch*	1990	draft	H. Wakabayashi, Tokyo University, Japan
FPC 840	GCA_900186575 (5)	Japan	*Plecoglossus altivelis*	1987	draft	H. Wakabayashi, Tokyo University, Japan
IT02	GCA_900186545 (5)	Italy	*Oncorhynchus mykiss*	2011	draft	A. Manfrin, Istituto Zooprofilattico Sperimentale delle Venezie, Adria, Italy
IT09	GCA_900186525 (5)	Italy	*Oncorhynchus mykiss*	2012	draft	A. Manfrin, Istituto Zooprofilattico Sperimentale delle Venezie, Adria, Italy
JIP 02/86	AM398681.2 (3)	France	*Oncorhynchus mykiss*	1986	complete	Culture collection of Unité de Virologie et Immunologie Moléculaires, INRA, Jouy-en-Josas, France
JIP 08/99	GCA_900186595 (5)	France	*Oncorhynchus mykiss*	1999	draft	Culture collection of Unité de Virologie et Immunologie Moléculaires, INRA, Jouy-en-Josas, France
JIP 16/00	GCA_900186515 (5)	France	*Oncorhynchus mykiss*	2000	draft	Culture collection of Unité de Virologie et Immunologie Moléculaires, INRA, Jouy-en-Josas, France
KU 051128-10	GCA_900186555 (5)	Japan	River Water	2005	draft	Culture collection of the Department of Fisheries, Kindai University, Nara, Japan
KU 060626-4	GCA_900186475 (5)	Japan	*Plecoglossus altivelis*	2006	draft	Culture collection of the Department of Fisheries, Kindai University, Nara, Japan
KU 060626-59	GCA_900186495 (5)	Japan	*Plecoglossus altivelis*	2006	draft	Culture collection of the Department of Fisheries, Kindai University, Nara, Japan
KU 061128-1	GCA_900186605 (5)	Japan	River Water	2006	draft	Culture collection of the Department of Fisheries, Kindai University, Nara, Japan
LM-01-Fp	GCA_900186685 (5)	Chile	*Oncorhynchus mykiss*	2006	draft	R. Avendaño-Herrera, Universidad Andrés Bello, Viña del Mar, Chile
LM-02-Fp	GCA_900186665 (5)	Chile	*Oncorhynchus mykiss*	2006	draft	R. Avendaño-Herrera, Universidad Andrés Bello, Viña del Mar, Chile
LVDJ XP189	GCA_900186645 (5)	France	*Tinca tinca*	1992	draft	M. Morand, Laboratoire Vétérinaire Départemental du Jura, Lons-le-Saulnier, France
NCIMB 1947^{T}	CP007207 (2)	USA	*Oncorhynchus kisutch*	Unknown	complete	National Collection of Industrial and Marine Bacteria, Aberdeen, UK
NO004	GCA_900186755 (5)	Norway	*Salmo trutta*	1998	draft	H. Nilsen, Norwegian Veterinary Institute, Norway
NO014	GCA_900186785 (5)	Norway	*Oncorhynchus mykiss*	2008	draft	H. Nilsen, Norwegian Veterinary Institute, Norway
NO042	GCA_900186825 (5)	Norway	*Salmo salar*	2008	draft	H. Nilsen, Norwegian Veterinary Institute, Norway
NO083	GCA_900186805 (5)	Norway	*Oncorhynchus mykiss*	2011	draft	H. Nilsen, Norwegian Veterinary Institute, Norway
NO098	GCA_900186815 (5)	Norway	*Salmo salar*	2011	draft	H. Nilsen, Norwegian Veterinary Institute, Norway
OSU THCO2-90	LT670843 (1)	USA	*Oncorhynchus kisutch*	1990	complete	M. Whipple, Oregon State University, Corvallis, USA
DIFR 950106-1/1	GCA_000767095.1 (4)	Denmark	*Oncorhynchus mykiss*	1995	complete	Culture collection of the Danish Institute for Fisheries Research, Frederiksberg, Denmark

(1) Rochat T, Barbier P, Nicolas P, Loux V, Pérez-Pascual D, Guijarro JA,Bernardet JF, Duchaud E. Genome Announc. 2017. Feb 23;5(8). pii: e01665-16. doi: 10.1128/genomeA.01665-16. PubMed PMID:28232446; PubMed Central PMCID: PMC5323625.

(2) Wu AK, Kropinski AM, Lumsden JS, Dixon B, MacInnes JI. Stand Genomic Sci. 2015 Jan 21;10:3. doi: 10.1186/1944-3277-10-3. eCollection 2015. PubMed PMID: 25685258; PubMed Central PMCID: PMC4322650.

(3) Duchaud E, Boussaha M, Loux V, Bernardet JF, Michel C, Kerouault B, Mondot S, Nicolas P, Bossy R, Caron C, Bessières P, Gibrat JF, Claverol S, Dumetz F, Le Hénaff M, Benmansour A. Nat Biotechnol. 2007 Jul;25(7):763-9. Epub 2007 Jun 24. PubMed PMID: 17592475.

(4) Castillo D, Christiansen RH, Dalsgaard I, Madsen L, Espejo R, Middelboe M. PLoS One. 2016 Apr 12;11(4):e0152515. doi: 10.1371/journal.pone.0152515. eCollection 2016. PubMed PMID: 27071075; PubMed Central PMCID: PMC4829187.

(5) E. Duchaud, T. Rochat, C. Habib, P. Barbier, V. Loux, C. Guérin, I. Dalsgaard, L. Madsen, H. Nilsen, T. Wiklund, K. Sundel, T. Wahli, N. Strepparava, A. Manfrin, G. Caburlotto, G. Wiens, E. Fujiwara-Nagata, R. Avendaño-Herrera, J-F. Bernardet & P. Nicolas. Front Microbiol. 2018 Feb 7;9:138. doi: 10.3389/fmicb.2018.00138. eCollection 2018. PMID:29467746. PMCID: PMC5808330.

Figure S1. Elastinolytic activity of the 34 isolates on TYES agar medium supplemented with 0.75% elastin.

Note the difference in halo size between isolates: most of the positive strains displayed a clearly visible halo whereas three strains (*i.e.*, FPC 840, NO004 and DK002) displayed almost no diffusion halo, elastin hydrolysis being restricted to underneath the bacterial growth.

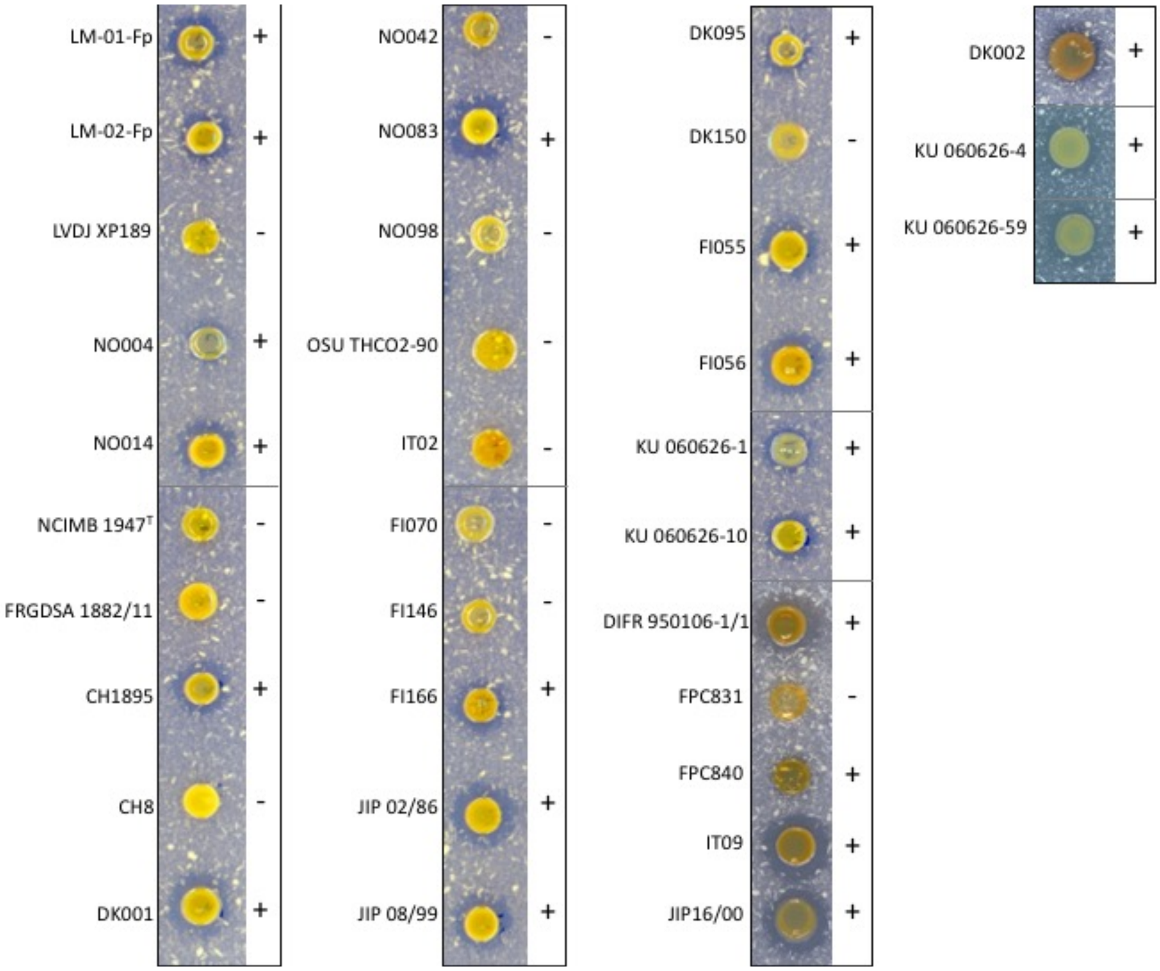

Figure S2. RT-PCR analysis of the elastase locus in strains JIP 02/86 and OSU THCO2-90.
A. Genomic map and primers sets used for the reverse transcriptase PCR.
Grey colored areas indicate conserved genomic regions between *F. psychrophilum* isolates. The elastase-coding gene (*FP0506*) is in black. The primers set used (Table 1) for overlapping PCR amplification (named F1 to F5) hybridize on JIP 02/86 or OSU THCO2-90 genomic sequences, as indicated in the figure (lines above and below the genomic map for JIP 02/86 and OSU THCO2-90, respectively). The theoretical results of PCR amplification are listed in the table. Black arrows indicate putative sequences corresponding to the consensus sequence of *Bacteroidetes* promoters (TAnnTTTG). No significant Rho-independent terminator was identified in these intergenic regions using the ARNold finding terminators software (http://rna.igmors.u-psud.fr/toolbox/arnold/).

Fragment	Primers sets	JIP 02/86	OSU THCO2-90
F1	TRO383+TRO442	0.73 kb	⦸
F2	TRO384+TRO388	1.0 kb	⦸
F3	TRO385+TRO389	1.40 kb	⦸
F4	TRO383+TRO389	2.10 kb	1.06 kb
F5	TRO387+TRO389	⦸	0.67 kb

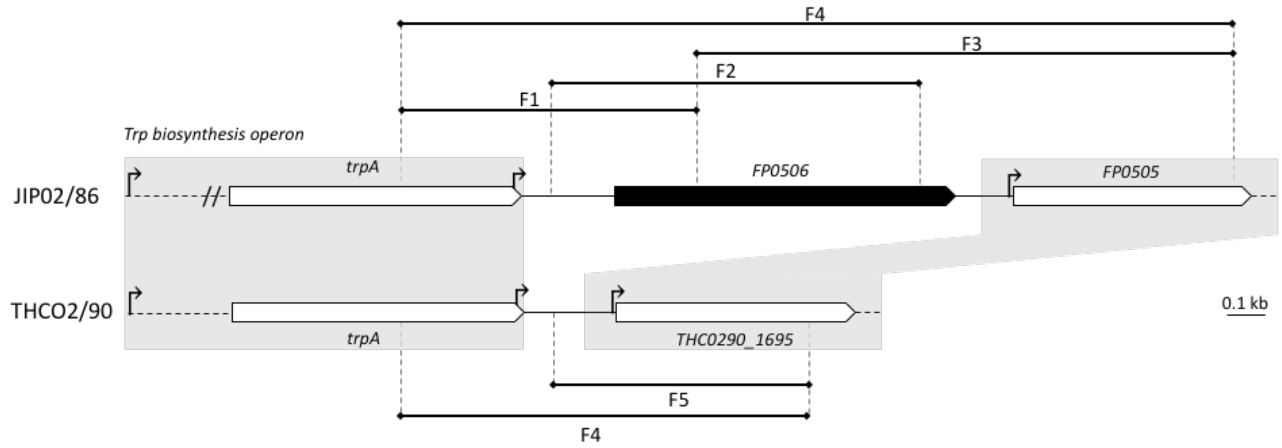

B. Agarose gel electrophoresis of the DNA products resulting from the RT-PCRs.
The PCR reactions were performed using the primer sets and cDNA synthetized by reverse transcriptase reaction (RT) using DNase-treated total RNA isolated from *F. psychrophilum* JIP 02/86 (left) and OSU THCO2-90 (right). Negative controls (-) correspond to PCR performed without reverse transcriptase and positive controls (+) to PCR performed using genomic DNA as matrix.

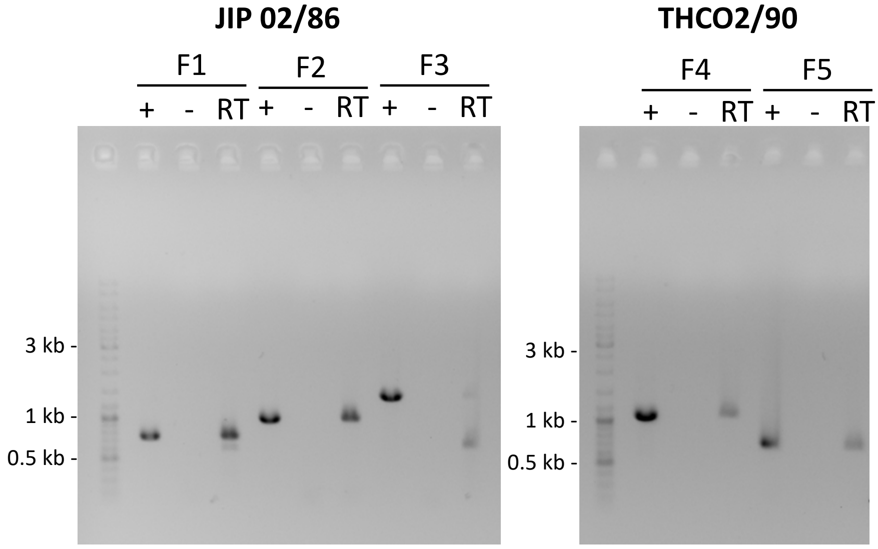

Figure S3. Plasmid map of pCP*Gm*r-FP0506.

Nucleotide sequence of the 1.4 kb-long region cloned into pCP*Gm*r and carrying the coding sequence of *FP0506* (red) flanked by 330 bp upstream and 170 bp downstream regions.

```
                                                                                        (-7) box
(FP0507)…GGAGCCATTATTGGAAGTGCTTTTATTAAAAATTTGACCGAAAATGGCGTTTCTAGCATTAGTAAATTTGTTTCAGAAA
TTTTTTAGagtaaaatttatgttaaatatttattaaataatacagttaaatactaatcttaaaacaaccaaacagtgtttttaccgat
aaaaacattcatcataccgattattaaacattaatgatagttttaaaaaaaacttttaaattattaataaaacacaaaattgttaaatt
taatataaaatttatatttttatcacgaaaaattaatcaaaccaacaatttaaaattaaccaacccaaaaattATGACAACAACCAAAA
AAACCAATTTATTTTTAACTGCATTAGCTTTAGGAATGTTTGCATCTTGTAGCAAAAACGACGATATCAAAACGATATCAACTGAAAAA
GCATTAGACCAACCCTCTTTACTAAACAAAGAATGTAGTTTTGTAGATAATAATTGGGCATCAGCTGCAACTTTATACACAACGTTACC
TAATTCTGGTAGTACTACAGGCGCAAGTTTGATGACAACTCAAAATTCTACAATAGCTTCTTTTTGGGGAAGAAGCGCTCCGTCTTTCC
GTTTTGTTAGAGATTTAACAACACCAAGTTCTACATTTAACGCAATTTCTTATAGTACAGGTAAAATTTATTTTGGAGAAGCAATTTTT
AAATGGGCATATAACAGAGACAATTCAAATCTAATTAATGTAATGATATTAGCTCATGAATACGGTCATCAATTACAATATGCTTTTGG
TTTGCCTTCTGTATCAGAAAGTACAGCAAGACCAAATGAACTGGAAGCTGACGGATTTTCAGGATACTATTTAAGAAGAGGATACGGTA
AATCTACGTTTGCTTCTATTGCGACAGCTTATGATGCTGCCTTTGCCATTGGCGACAATAATGTAACAAGTCCAGGTCATCATGGCACA
CCAGCACAAAGAAGATCTGCTGTACGTTTAGGTTTCTTATTAGCAGACCCAACAAATGCAAAATTAACAGCTTCGGCTTTTGATTCTAA
TTTCTTTTACTACTATACAGGTGTAAAAAACGGAACATATCGCCAAGCTAAACCAGCTAATTTTGACGCAAAAATGGATGCGAATATTA
AAATACATTCAGACGAATTAAGAAGAATTCAATCTGGAGAAATGTCTGATCAAGAATACTTTAATCTACAATAACAAACAAAAAACTAT
TTTTTTAAAGCTACTTTTTTATAAAGTAGCTTTTTTTATTCATTTTTATTAACTGTTAAAAAAATGTTAATTAAAAACTAAACAAACGT
TTAATTTAAACAATTGTTTAAATTTGTATCGATTTTAAAAATCAATACCCATGACCGATTTTAATG
```

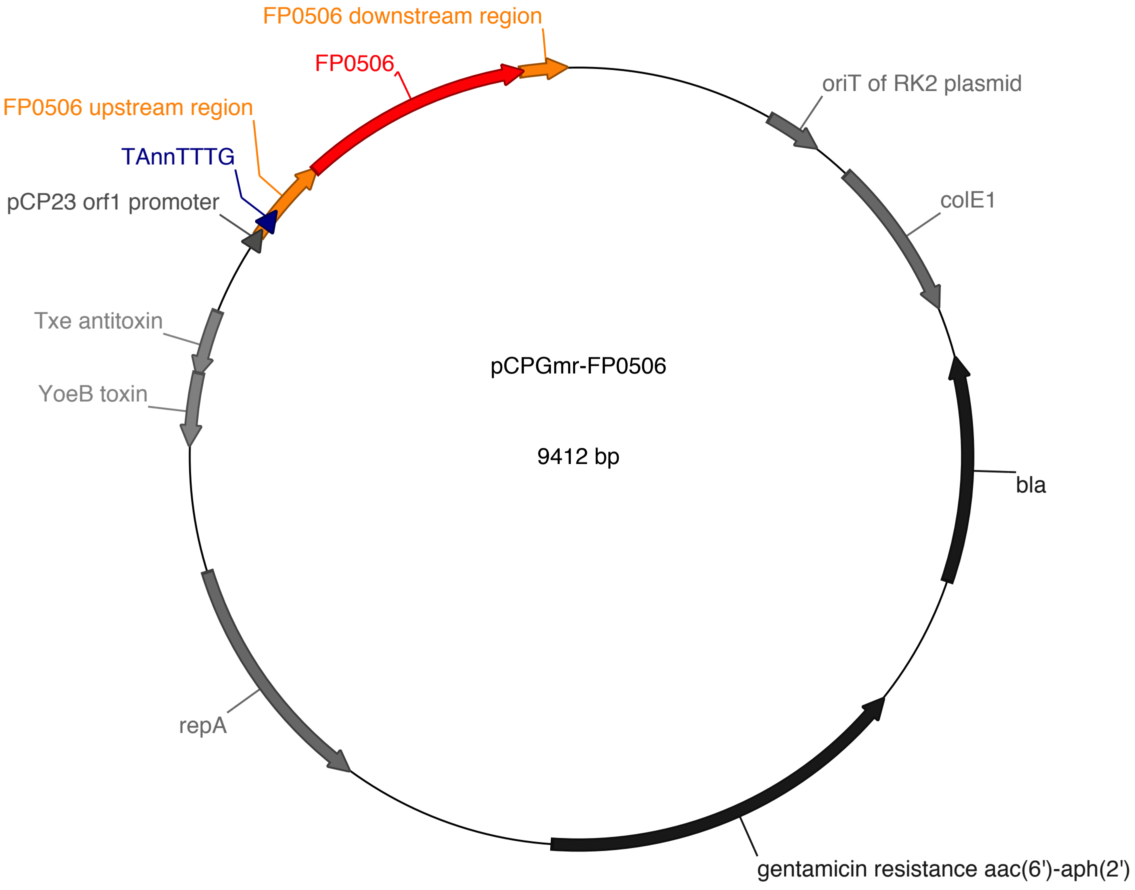

Figure S4. Predicted protein domains and important amino-acid residues in FP0506

```
MTTTKKTNLFLTALALGMFASCSKNDDIKTISTEKALDQPSLLNKECSFVDNNWASAATL

61
YTTLPNSGSTTGASLMTTQNSTIASFWGRSAPSFRFVRDLTTPSSTFNAISYSTGKIYFG

121                        (HExxH)                   (E/HxxZ)
EAIFKWAYNRDNSNLINVMILAHEYGHQLQYAFGLPSVSESTARPNELEADGFSGYYLRR

181
GYGKSTFASIATAYDAAFAIGDNNVTSPGHHGTPAQRRSAVRLGFLLADPTNAKLTASAF

241
DSNFFYYYTGVKNGTYRQAKPANFDAKMDANIKIHSDELRRIQSGEMSDQEYFNLQ
```

Highlighted in grey (position 1-21), the predicted lipoprotein signal peptide; highlighted in cyan (position 22), the lipidated cysteine residue corresponding to the +1 amino-acid of the mature protein; highlighted in green the predicted lipoprotein export signal (LES) corresponding to the +3, +4, +5 and +6 amino-acid of the mature protein; and highlighted in yellow, the Jongeneel consensus. In green, the 4 predicted metal binding amino acids: two histidines belonging to the HExxH motif (positions 143-147), one glutamate (position 167) belonging to the E/HxxZ motif and the last histidine, position 211. In bold, the alanine residue (position 170), encompassed in the ExxA motif specific of the Gluzincins clan. In blue, the other catalytic residue (glutamic acid position 144) in addition to the four metal ligands.

Figure S5. Expression of *FP0506* in a *F. psychrophilum* mutant deficient for exoproteolytic activity.

The pCP*Gm*[r]-*FP0506* plasmid was introduced by conjugation into the transposition mutant *gldG*::Tn4351 previously characterized (Pérez-Pascual D. *et al*, 2017). This mutant was shown to be deficient in exoproteolytic activity probably due to an indirect effect on the Type IX secretion system. In this mutant, no protein degradation is detectable around bacterial cells on TYES agar supplemented with casein or gelatin as a substrate. However, the expression of *FP0506* results in a clearing area on TYES agar supplemented with 0.75% elastin similar to the wild-type strain and the secretion-deficient mutant. The diffusion of the enzyme may result from other parameters such as partial cell lysis into bacterial colonies or secretion of outer membrane vesicles.

Pérez-Pascual D, Rochat T, Kerouault B, Gomez E, Neulat-Ripoll F, Henry C, Quillet E, Guijarro JA, Bernardet JF, Duchaud E. 2017. More Than Gliding: Involvement of GldD and GldG in the Virulence of *Flavobacterium psychrophilum*. Front Microbiol 8:2168.

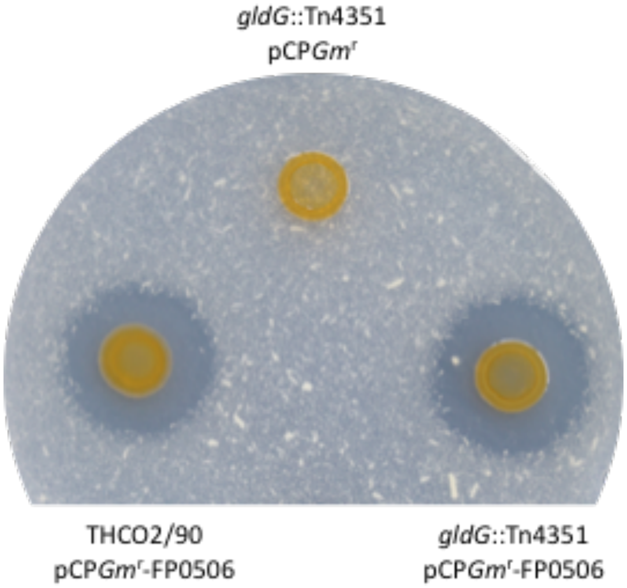

Figure S6. Pairwise alignment of the mature FP0506 and PDB-3C37 obtained using HHpred.

The sequence of FP0506 was used after signal peptide removal to increase signal/noise ratio against the three-dimensional the protein structures Protein Data Bank (PDB_mmCIF70_28_Jul) with HHpred. The alignment with the first hit 3C37 is presented. Important residues are boxed (the numbering from UniProtKB accession Q74D82, corresponding to the primary sequence of PDB-3C37, is indicated).

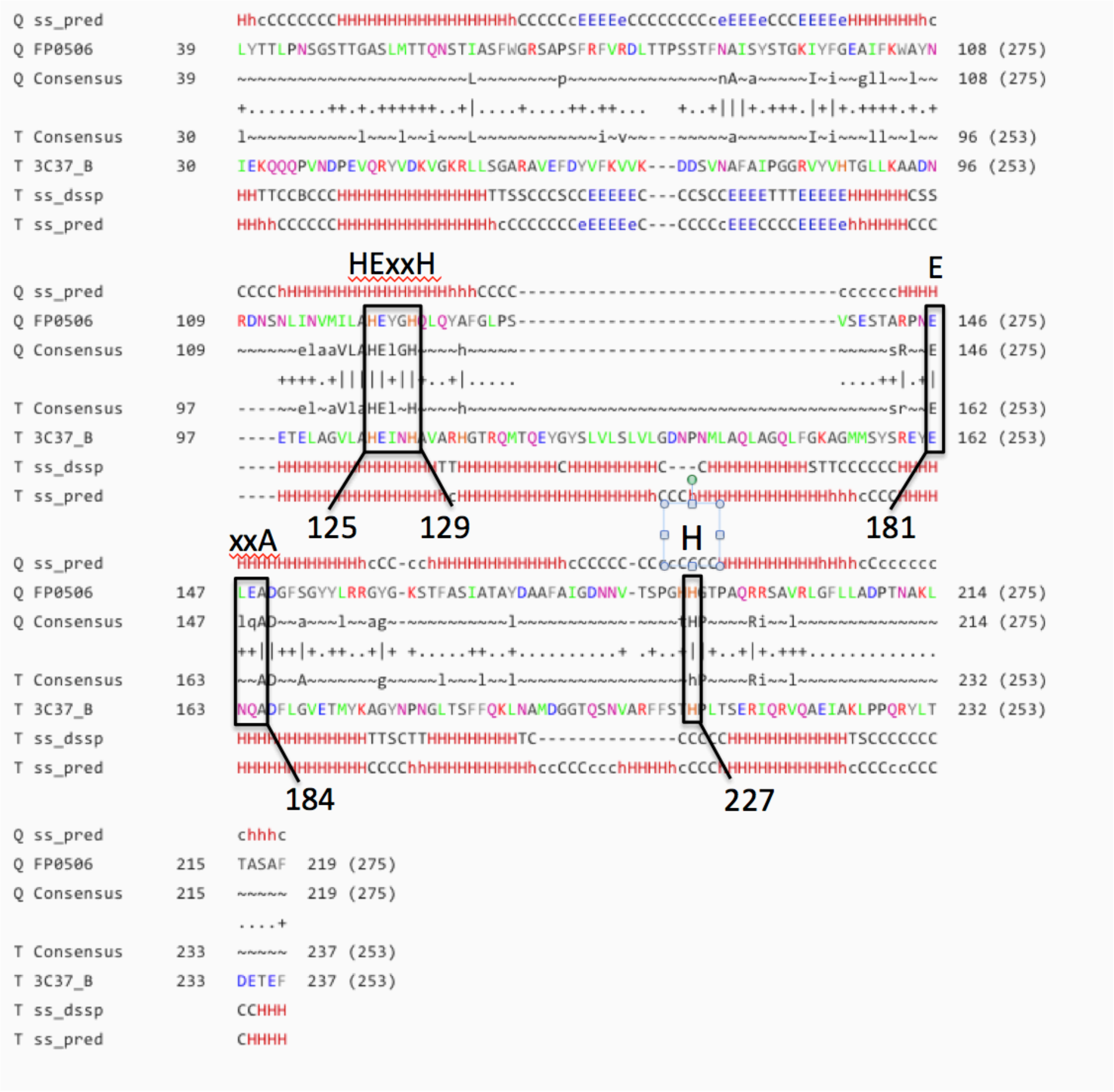

Figure S7. A) Predicted local quality score of FP0506 using the SwissModel and PDB-3C37 as a template.

Local structural reliability score (QMEAN), computed for each residues. Local QMEAN is in the range [0,1] with one being good. Typically, residues showing a score below 0.6 are expected to be of low quality; this threshold is shown by the black dotted line. All residues below this threshold are in black in figure 3. The two main regions below this threshold correspond to the two loops with many black residues in figure 3.

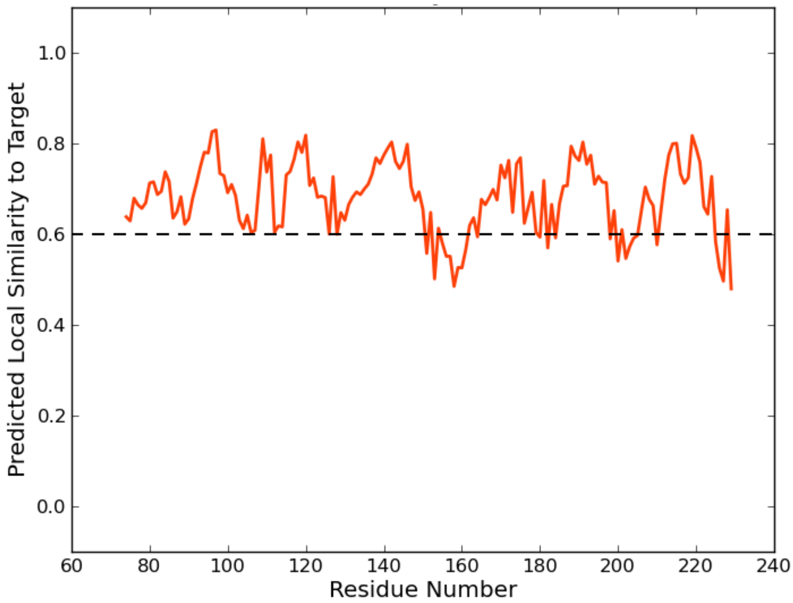

S7. B) Superimposition of the model calculated by SwissModel for FP0506 (in blue) with the reference resolved structure of PDB-3C37 (in magenta).

Because of the weak similarities, the unaligned N-terminal and C-terminal regions of FP0506 (*i.e.*, residues 1 to 74 and 229 to 296, respectively) were not included in the model.

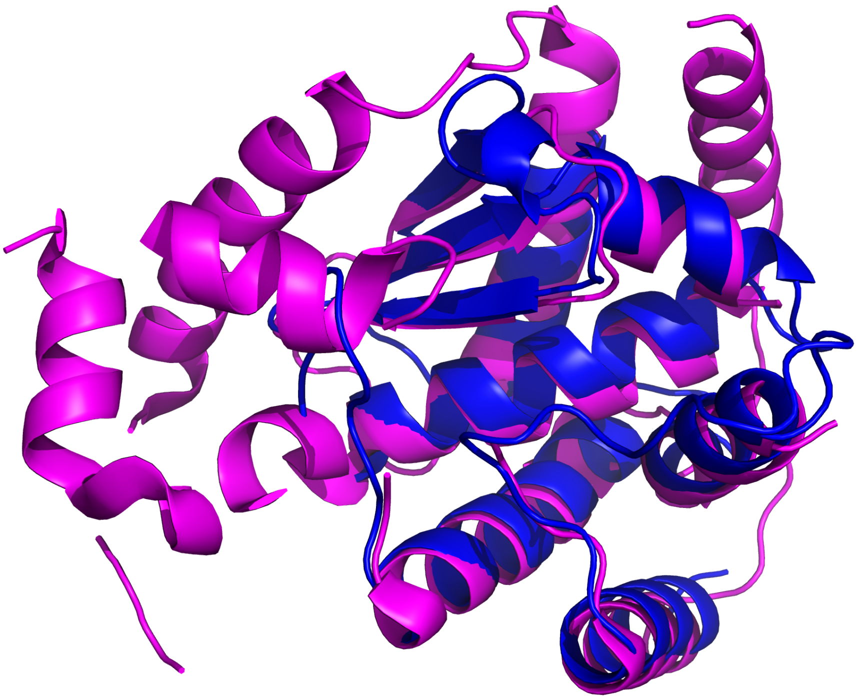

Figure S8. Phylogenetic tree of FP0506 homologous proteins

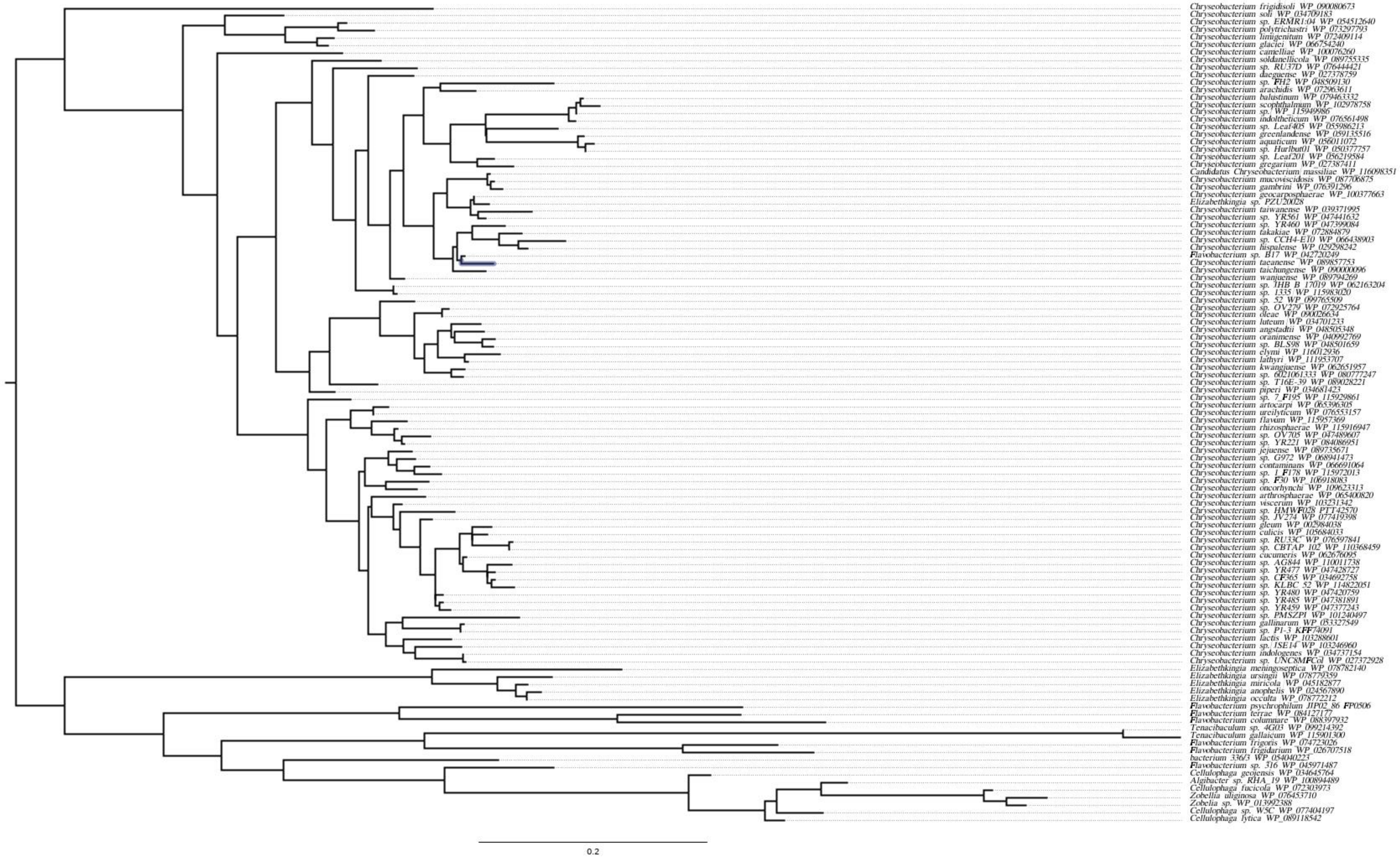

Supplement: Supplemental file 1 [file AEM.02535-18-s0001.pdf]
